# Multiple Machine Learning Algorithms for Predicting Postoperative Residual Tumor in Pituitary Neuroendocrine Tumors

**DOI:** 10.1002/cns.71244

**Published:** 2026-11-02

**Authors:** Mingrui Li, Chenyang Zhao, Guodong Tang, Yang Li, Qing Liu

**Affiliations:** ^1^ Department of Neurosurgery Xiangya Hospital Central South University Changsha China

**Keywords:** machine learning, pituitary neuroendocrine tumor, postoperative residual tumor, precision neurosurgery, predictive model

## Abstract

**Aims:**

To develop and validate an interpretable machine learning (ML) model for predicting radiologically confirmed postoperative residual tumor (RT) in pituitary neuroendocrine tumors (PitNETs).

**Methods:**

We retrospectively analyzed 584 surgically treated patients with PitNETs from a single center, using a random 7:3 training–test split and internal cross‐validation. The primary endpoint was radiologically confirmed postoperative RT, assessed using MRI obtained within 48 h after surgery and longitudinal follow‐up MRI. Candidate variables were selected using LASSO regression, with nine ML algorithms developed. Discrimination, calibration, clinical utility, interobserver agreement, and SHAP‐based interpretability were assessed.

**Results:**

Postoperative RT occurred in 72 patients (12.3%). Sensitivity analysis showed high agreement between 48‐h MRI and follow‐up confirmation (96.4%; Cohen's kappa = 0.837). Interobserver agreement was high for retrosellar invasion (kappa = 0.825), internal carotid artery invasion (kappa = 0.960), and Knosp grade (quadratic weighted kappa = 0.994). Eight predictors were retained: maximum sagittal diameter, maximum coronal diameter, age, Knosp grade, internal carotid artery invasion, sphenoid sinus pneumatization, tumor consistency, and retrosellar invasion. In the internal test set, the random forest model achieved high discrimination (AUC = 0.945; accuracy = 0.938), with performance comparable to LightGBM and XGBoost in DeLong testing. LightGBM had the lowest Brier score.

**Conclusion:**

Random forest was selected for the web tool after internal evaluation, with discrimination comparable to LightGBM, XGBoost, and SVM. Its generalizability to other centers and future patient cohorts remains unproven because evaluation used only a single‐center random split and internal cross‐validation. External and temporal validation with calibration assessment is needed before clinical implementation.

## Graphical abstract

An explainable random forest–based machine learning model accurately predicted postoperative residual tumors in PitNETs, with tumor size and Knosp grade as major predictors, offering a web‐based tool for individualized surgical risk assessment and precision management.

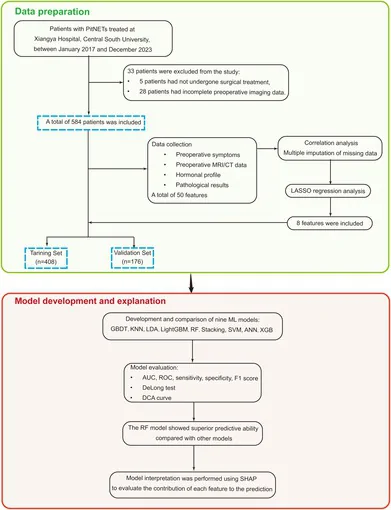

## 1 Introduction

Pituitary neuroendocrine tumors (PitNETs) are the most common tumors originating in the sellar region, accounting for approximately 15% of central nervous system neoplasms [1]. PitNETs arise from pituitary glandular cells and are classified as functional or nonfunctional depending on hormone secretion. Functional tumors present distinct clinical manifestations depending on the secreted hormone. Prolactin‐ or gonadotropin‐secreting tumors may cause menstrual disorders and sexual dysfunction; growth hormone‐secreting tumors lead to gigantism or acromegaly; adrenocorticotropic hormone‐secreting tumors result in Cushing's disease; and the rare thyrotropin‐secreting subtype induces central hyperthyroidism [2]. Nonfunctional PitNETs predominantly present with mass effect, often manifesting as visual deterioration and headache [3]. PitNETs are further divided into microadenomas (< 10 mm maximum diameter) and macroadenomas (≥ 10 mm). Approximately 10% of people harbor incidental PitNETs identified during imaging or autopsy, of which 99.9% are microadenomas. Symptomatic PitNETs affect about 1/1100 individuals, who mostly present with macroadenomas. Prolactinomas can be managed medically using dopamine agonists, but surgery remains the first‐line treatment for most PitNETs [3]. Although PitNETs are generally benign, their anatomical location and occasional invasive behavior complicate surgical management. Residual tumors are associated with persistent endocrine dysfunction and with increased progression and recurrence, necessitating adjuvant therapy or long‐term surveillance [4]. Early prediction of postoperative residual tumor (RT) is critical for surgical planning and personalized postoperative management.

Traditional statistical approaches such as logistic regression have long been used for risk stratification and prognostic modeling in tumors [5]. However, they rely on rigid linear assumptions and are limited in addressing complex nonlinear interactions. In contrast, machine learning (ML) techniques can identify latent patterns and deliver superior predictive performance [6, 7]. In neuro‐oncology, ML is used for survival prediction, radiogenomic correlation, tumor classification, and outcome estimation [8]. Previous studies have focused on integrating radiological features to predict tumor subtypes and postoperative complications [9, 10, 11, 12, 13]. Studies have applied ML algorithms to predict surgical outcomes and recurrence risks in PitNET [14, 15]. These approaches consistently outperform traditional methods, highlighting ML's potential to enhance clinical decision‐making.

Despite their predictive power, ML algorithms are often criticized for the internal decision‐making process remaining opaque. To mitigate this, explainable artificial intelligence methods have been developed. Among them, SHapley Additive exPlanations (SHAP) uses a game‐theoretic foundation and capacity to assign consistent, locally accurate contribution values to each feature [16]. Interpretability is crucial in medicine, fostering trust in ML predictions and supporting evidence‐based integration. SHAP visualizations further facilitate interdisciplinary communication by providing transparent insights into predictive factors.

The present study aimed to develop an ML‐based model for predicting postoperative PitNET RT. By incorporating SHAP, our approach achieved high predictive accuracy and interpretable visualizations illustrating the contribution of each variable to predictions. This emphasis on accuracy and transparency aligns with the principles of precision medicine, prioritizing personalized, data‐driven decision support.

## 2 Methods

### 2.1 Data Collection

We retrospectively collected clinical data of patients with PitNETs treated at Xiangya Hospital of Central South University between January 2017 and December 2023. The inclusion criteria were: (1) clinical PitNET diagnosis, confirmed by immunohistochemistry according to the 2022 World Health Organization classification; (2) age > 18 years; (3) complete medical records, including symptoms and preoperative and postoperative hormone levels; (4) complete imaging data, with preoperative cranial MRI performed at our institution; (5) surgery performed by the same neurosurgeon. The exclusion criteria were: (1) no surgery (*n* = 5); and (2) missing clinical data (*n* = 28). Ultimately, 584 patients were included.

All variables were extracted from electronic medical records, including demographic information, preoperative symptoms, preoperative MRI tumor characteristics, surgical variables, pathological results, postoperative complications, and hormone levels. Hormonal abnormalities were defined as: (1) secondary hypothyroidism: thyroid stimulating hormone < 0.27 mIU/L and free thyroxine < 11 pmol/L; (2) hyperprolactinemia: prolactin > 15.2 μg/L [17]; (3) growth hormone excess: growth hormone > 0.47 nmol/L; (4) adrenal insufficiency: morning serum cortisol < 50 nmol/L with normal or reduced adrenocorticotropic hormone levels. When serum cortisol levels were 5–18 ng/dL, a hypoglycemia stimulation test was performed; if stimulated cortisol remained ≤ 5 ng/dL, adrenal insufficiency was confirmed [18].

The primary endpoint was radiologically confirmed postoperative RT. Postoperative hormonal status and used for descriptive and exploratory analyses, hormonal persistence or remission was not included in the endpoint definition. Postoperative RT was determined based on longitudinal radiological assessment. Early postoperative contrast‐enhanced MRI within 48 h after surgery was used as the initial assessment of the resection extent. Postoperative follow‐up MRI at 3 months, 6 months, 1 year, or later was also reviewed. Persistent nodular or mass‐like enhancement in the sellar, parasellar, suprasellar, or retrosellar region on serial follow‐up MRI was considered confirmed RT, whereas transient linear or diffuse enhancement that disappeared or decreased during follow‐up was not classified as RT. Complete resection was defined as the absence of RT; near‐total and sub‐total resection were defined as RT with a maximum diameter ≤ 3 and > 3 mm, respectively, with both classified as RT [19]. Imaging was independently assessed by two senior neurosurgeons blinded to predictive factors, with consultation with a third blinded neurosurgeon until final consensus, if necessary.

To evaluate the robustness of the endpoint definition, a sensitivity analysis compared the initial 48‐h MRI‐based classification with the final longitudinal MRI‐confirmed classification. Overall agreement and Cohen's kappa were calculated. Interobserver agreement for radiological predictors including retrosellar invasion, ICA invasion, and Knosp grade was assessed using Cohen's kappa; quadratic weighted Cohen's kappa was used for ordinal Knosp grade.

### 2.2 Data Processing and ML

Data preprocessing and variable selection were conducted using R (version 4.4.0). Baseline characteristics were summarized using the “compareGroups” package, with postoperative RT status as the grouping variable. Missing continuous variables were imputed using multiple imputation (“mice” package, *m* = 5). Continuous variables following a normal distribution are expressed as mean ± standard deviation and were compared with the independent‐sample *t*‐test; non‐normally distributed data are expressed as median (interquartile range) and were compared using the Mann–Whitney *U*‐test. Categorical variables are summarized as frequencies and percentages and were compared using Pearson's chi‐squared test. Least absolute shrinkage and selection operator (LASSO) regression was applied to identify predictors most relevant to postoperative RT status.

Subsequently, nine ML models were developed in Python (version 3.10.0) to predict postoperative RT: Gradient Boosting Decision Tree, *k*‐Nearest Neighbor, Linear Discriminant Analysis, Light Gradient Boosting Machine (LightGBM), Random Forest (RF), Stacking, Support Vector Machine (SVM), Artificial Neural Network, and eXtreme Gradient Boosting (XGBoost). Patients were randomly split into training and validation cohorts at a 7:3 ratio. To address class imbalance, SMOTE oversampling was performed. Model optimization was conducted using 5 × 5‐fold cross‐validation and grid search on the optimal feature subset to obtain the final hyperparameters for each model. Each model was then retrained on the training cohort using the optimal feature subset and tuned parameters. SMOTE oversampling was applied only within the training cohort and cross‐validation process; the held‐out validation cohort was kept in its original class distribution for unbiased performance evaluation.

Model performance was assessed using the area under the receiver operating characteristic curve (AUC), sensitivity, specificity, accuracy, and F1 score. Calibration curves were plotted to assess agreement between predicted probabilities and observed outcomes. DeLong's test was used to compare AUCs between models. Decision curve analysis was conducted to evaluate the clinical net benefit across a range of threshold probabilities. The best‐performing model was selected based on training and validation performance.

For comparison, we evaluated a logistic regression model using Knosp grade, maximum sagittal and coronal tumor diameters, and ICA invasion. We assessed its AUC and Brier score in the held‐out test set and evaluated net benefit using decision curve analysis. Exploratory subgroup analyses assessed performance in functional and nonfunctional PitNETs and in giant (maximum diameter ≥ 40 mm) and non‐giant adenomas.

Model selection considered the balance of discrimination, calibration, decision‐curve performance, and interpretability. Statistically non‐significant differences in AUC were not interpreted as evidence of model superiority. To elucidate the contribution of individual features to model predictions, we employed SHAP using the background dataset of the original non‐SMOTE training samples to avoid shifting the expected value toward the artificially balanced class distribution. SHAP values were calculated for all cases in the validation set, providing local and global interpretability and enhancing model transparency and clinical interpretability.

## 3 Results

### 3.1 Baseline Clinical Information

In total, 584 patients were included, with 512 classified into the total tumor resection (TTR) group, and 72 into the RT group (Table 1). There were significant differences between groups in multiple clinical features (*p* < 0.001). The RT group had significantly larger maximum tumor diameter than the TTR group (40.2 vs. 25.9 mm). Maximum coronal (34.7 vs. 22.6 mm), sagittal (31.6 vs. 19.4 mm), and vertical diameters (35.6 vs. 23.9 mm) were also larger. Tumor invasion into the retrosellar region was more frequent in the RT group than in the TTR group (31.9% vs. 6.05%). Indicators reflecting invasion of bilateral anatomical structures, including cavernous sinus involvement, internal carotid artery (ICA) invasion, and Knosp grade also showed significant differences between groups. Additionally, tumor consistency differed between groups. No clear relationship was observed between pre‐ and post‐operative Day 1 hormone levels and the extent of tumor resection.

**TABLE 1:** Comparison of clinical characteristics, imaging features, and other variables between the TTR group and the RT group.

	ALL, *N* = 584	TTR Group, *N* = 512	RT Group, *N* = 72	*p* overall
Sex				
Female	310 (53.1%)	275 (53.7%)	35 (48.6%)	0.493
Male	274 (46.9%)	237 (46.3%)	37 (51.4%)	
Age	48.4 (13.0)	48.6 (13.0)	46.4 (13.4)	0.197
History of bromocriptine use				
No	546 (93.5%)	475 (92.8%)	71 (98.6%)	0.071
Yes	38 (6.51%)	37 (7.23%)	1 (1.39%)	
No symptom				
No	522 (89.4%)	457 (89.3%)	65 (90.3%)	0.953
Yes	62 (10.6%)	55 (10.7%)	7 (9.72%)	
Dizzy				
No	447 (76.5%)	385 (75.2%)	62 (86.1%)	0.058
Yes	137 (23.5%)	127 (24.8%)	10 (13.9%)	
Headache				
No	371 (63.5%)	326 (63.7%)	45 (62.5%)	0.950
Yes	213 (36.5%)	186 (36.3%)	27 (37.5%)	
Visual deterioration				
No	271 (46.4%)	249 (48.6%)	22 (30.6%)	0.036
Left	56 (9.59%)	46 (8.98%)	10 (13.9%)	
Right	43 (7.36%)	36 (7.03%)	7 (9.72%)	
Both	214 (36.6%)	181 (35.4%)	33 (45.8%)	
Visual field defect				
No	484 (82.9%)	430 (84.0%)	54 (75.0%)	0.100
Left	27 (4.62%)	20 (3.91%)	7 (9.72%)	
Right	26 (4.45%)	23 (4.49%)	3 (4.17%)	
Both	47 (8.05%)	39 (7.62%)	8 (11.1%)	
Diplopia				
No	550 (94.2%)	483 (94.3%)	67 (93.1%)	0.595
Yes	34 (5.82%)	29 (5.66%)	5 (6.94%)	
Tosis				
No	573 (98.1%)	504 (98.4%)	69 (95.8%)	0.172
Left	7 (1.20%)	5 (0.98%)	2 (2.78%)	
Right	4 (0.68%)	3 (0.59%)	1 (1.39%)	
Oculomotor dysfunction				
No	577 (98.8%)	506 (98.8%)	71 (98.6%)	0.435
Left	2 (0.34%)	2 (0.39%)	0 (0.00%)	
Right	2 (0.34%)	1 (0.20%)	1 (1.39%)	
Both	3 (0.51%)	3 (0.59%)	0 (0.00%)	
Dysosmia				
No	574 (98.3%)	505 (98.6%)	69 (95.8%)	0.114
Yes	10 (1.71%)	7 (1.37%)	3 (4.17%)	
Diabetes insipidus				
No	546 (93.5%)	479 (93.6%)	67 (93.1%)	0.801
Yes	38 (6.51%)	33 (6.45%)	5 (6.94%)	
Amenorrhea/galactorrhea				
No	497 (85.1%)	435 (85.0%)	62 (86.1%)	0.936
Yes	87 (14.9%)	77 (15.0%)	10 (13.9%)	
Acromegaly				
No	513 (87.8%)	446 (87.1%)	67 (93.1%)	0.210
Yes	71 (12.2%)	66 (12.9%)	5 (6.94%)	
Cushing's syndrome				
No	559 (95.7%)	488 (95.3%)	71 (98.6%)	0.346
Yes	25 (4.28%)	24 (4.69%)	1 (1.39%)	
Hypothyroidism				
No	568 (97.3%)	498 (97.3%)	70 (97.2%)	1.000
Yes	16 (2.74%)	14 (2.73%)	2 (2.78%)	
Sexual dysfunction				
No	550 (94.2%)	483 (94.3%)	67 (93.1%)	0.595
Yes	34 (5.82%)	29 (5.66%)	5 (6.94%)	
Diabetes mellitus				
No	529 (90.6%)	465 (90.8%)	64 (88.9%)	0.757
Yes	55 (9.42%)	47 (9.18%)	8 (11.1%)	
Pituitary apoplexy				
No	473 (81.0%)	414 (80.9%)	59 (81.9%)	0.953
Yes	111 (19.0%)	98 (19.1%)	13 (18.1%)	
Tumor form				
Round	267 (45.7%)	249 (48.6%)	18 (25.0%)	< 0.001
Dumbbell‐shaped	87 (14.9%)	77 (15.0%)	10 (13.9%)	
Lobulated	28 (4.79%)	17 (3.32%)	11 (15.3%)	
Irregular	202 (34.6%)	169 (33.0%)	33 (45.86%)	
Tumor size				
Microadenoma (< 10 mm in diameter)	25 (4.28%)	24 (4.69%)	1 (1.39%)	< 0.001
Macroadenoma (≥ 10 mm in diameter)	409 (70.0%)	378 (73.8%)	31 (43.1%)	
Giant pituitary adenoma (≥ 40 mm in diameter)	150 (25.7%)	110 (21.5%)	40 (55.6%)	
Maxdiameter (mm)	27.7 (12.5)	25.9 (10.9)	40.2 (15.6)	< 0.001
Maximum coronal length (mm)	24.1 (10.3)	22.6 (8.66)	34.7 (14.1)	< 0.001
Maximum sagittal length (mm)	20.9 (10.3)	19.4 (8.61)	31.6 (14.5)	< 0.001
Maximum height (mm)	25.3 (12.2)	23.9 (11.2)	35.6 (14.3)	< 0.001
Intrasellar invasion				
No	4 (0.68%)	3 (0.59%)	1 (1.39%)	0.410
Yes	580 (99.3%)	509 (99.4%)	71 (98.6%)	
Suprasellar invasion				
No	174 (29.8%)	162 (31.6%)	12 (16.7%)	0.014
Yes	410 (70.2%)	350 (68.4%)	60 (83.3%)	
Sphenoid sinus invasion				
No	460 (78.8%)	412 (80.5%)	48 (66.7%)	0.011
Yes	124 (21.2%)	100 (19.5%)	24 (33.3%)	
Retrosellar invasion				
No	530 (90.8%)	481 (93.9%)	49 (68.1%)	< 0.001
Yes	54 (9.25%)	31 (6.05%)	23 (31.9%)	
Optic chiasm compression				
No	153 (26.2%)	139 (27.1%)	14 (19.4%)	0.212
Yes	431 (73.8%)	373 (72.9%)	58 (80.6%)	
ICA invasion				
No	370 (63.4%)	354 (69.1%)	16 (22.2%)	< 0.001
Left	83 (14.2%)	61 (11.9%)	22 (30.6%)	
Right	61 (10.4%)	50 (9.77%)	11 (15.3%)	
Both	70 (12.0%)	47 (9.18%)	23 (31.9%)	
Knosp Grade				
0	75 (12.8%)	72 (14.1%)	3 (4.17%)	< 0.001
1	132 (22.6%)	127 (24.8%)	5 (6.94%)	
2	146 (25.0%)	139 (27.1%)	7 (9.72%)	
3	128 (21.9%)	105 (20.5%)	23 (31.9%)	
4	103 (17.6%)	69 (13.5%)	34 (47.2%)	
Sphenoid sinus pneumatization				
Conchal type	1 (0.17%)	1 (0.20%)	0 (0.00%)	
Presellar type	16 (2.74%)	10 (1.95%)	6 (8.33%)	
Semisellar type	45 (7.71%)	43 (8.40%)	2 (2.78%)	
Sellar type	117 (20.0%)	108 (21.1%)	9 (12.5%)	
Postsellar type	331 (56.7%)	300 (58.6%)	31 (43.1%)	
Sphenoid sinus invasion	56 (9.59%)	43 (8.40%)	13 (18.1%)	
Postoperative change	18 (3.08%)	7 (1.37%)	11 (15.3%)	
Surgical approach				
Endoscopic transsphenoidal surgery	56 (9.59%)	45 (8.79%)	11 (15.3%)	< 0.001
Microscopic transsphenoidal surgery	473 (81.0%)	433 (84.6%)	40 (55.6%)	
Craniotomy	54 (9.25%)	33 (6.45%)	21 (29.2%)	
Combined transcranial–endonasal approach	1 (0.17%)	1 (0.20%)	0 (0.00%)	
Third ventricle invasion				
No	553 (94.7%)	491 (95.9%)	62 (86.1%)	0.002
Yes	31 (5.31%)	21 (4.10%)	10 (13.9%)	
Tumor texture				
Soft	517 (88.5%)	465 (90.8%)	52 (72.2%)	< 0.001
Firm	67 (11.5%)	47 (9.18%)	20 (27.8%)	
Blood loss (mL)	141 (204)	127 (188)	239 (276)	0.001
Autologous fascial repair				
No	573 (98.1%)	505 (98.6%)	68 (94.4%)	0.036
Yes	11 (1.88%)	7 (1.37%)	4 (5.56%)	
CSF leak				
No	565 (96.7%)	495 (96.7%)	70 (97.2%)	1.000
Yes	19 (3.25%)	17 (3.32%)	2 (2.78%)	
Pathological classification				
Subtype not documented	121 (20.7%)	107 (20.9%)	14 (19.4%)	
Lactotroph PitNET	67 (11.5%)	55 (10.7%)	12 (16.7%)	
Corticotroph PitNET	40 (6.85%)	36 (7.03%)	4 (5.56%)	
Gonadotroph PitNET	142 (24.3%)	130 (25.4%)	12 (16.7%)	
Thyrotroph PitNET	3 (0.51%)	2 (0.39%)	1 (1.39%)	
Somatotroph PitNET	23 (3.94%)	23 (4.49%)	0 (0.00%)	
Plurihormonal PitNET	40 (6.85%)	33 (6.45%)	7 (9.72%)	
Nonfunctioning PitNET	148 (25.3%)	126 (24.6%)	22 (30.6%)	
FT3 (pmol/L)	4.30 (1.49)	4.31 (1.24)	4.23 (2.66)	0.810
FT4 (pmol/L)	14.1 (4.69)	14.1 (4.27)	13.5 (7.03)	0.464
TSH (μIU/L)	2.61 (6.52)	2.65 (6.93)	2.32 (1.78)	0.370
FSH (IU/L)	16.1 (22.4)	15.9 (21.9)	17.3 (26.1)	0.675
LH (IU/L)	7.64 (9.47)	7.85 (9.63)	6.18 (8.18)	0.116
PRL (ng/mL)	51.9 (93.0)	52.8 (92.9)	45.9 (94.5)	0.565
GH (ng/mL)	5.04 (15.0)	5.19 (15.4)	3.97 (11.6)	0.423
ACTH (pmol/L)	10.7 (13.0)	10.6 (13.3)	11.2 (10.3)	0.646
Cortisol (nmol/L)	226 (232)	226 (237)	222 (199)	0.878

Abbreviations: ACTH, Adrenocorticotropic Hormone; CSF, Cerebrospinal Fluid; FSH, Follicle‐Stimulating Hormone; FT3, Free Triiodothyronine; FT4, Free Thyroxine; GH, Growth Hormone; LH, Luteinizing Hormone; PitNET, Pituitary Neuroendocrine Tumor; PRL, Prolactin; RT, Residual Tumor; TSH, Thyroid‐Stimulating Hormone; TTR, Total Tumor Resection.

### 3.2 Sensitivity Analysis of RT Classification

To address the influence of early postoperative imaging changes on RT classification, we compared initial 48‐h MRI assessments with final longitudinal radiological assessments using serial follow‐up MRI (Table S1). Among all patients, 563 showed concordant classification between 48‐h MRI and final follow‐up confirmation, yielding an agreement of 96.4% and a Cohen's kappa of 0.837. Twenty‐one patients (3.6%) were reclassified after follow‐up review. Specifically, 12 initially suspected as RT on 48‐h MRI were reclassified as non‐RT; early postoperative enhancement may have reflected hematoma, inflammatory changes, or reactive tissue changes rather than true RT. Conversely, 9 patients initially classified as non‐RT were reclassified as RT during follow‐up. The final RT definition was robust and was not materially biased by early postoperative MRI changes.

### 3.3 Interobserver Agreement of Key Radiological Predictors

Interobserver agreement was excellent for the principal radiological variables used in model development (Table S2). The observed agreement was 96.9% for retrosellar invasion (Cohen's kappa, 0.825; 95% CI, 0.740–0.900; *p* < 0.001). Agreement was 97.8% for ICA invasion (Cohen's kappa, 0.960; 95% CI, 0.936–0.980; *p* < 0.001). For Knosp grade, quadratic weighted Cohen's kappa reached 0.994 (95% CI, 0.990–0.997; *p* < 0.001), indicating highly reproducible ordinal grading between observers.

### 3.4 Selection of Predictor Variables

In the entire cohort, we investigated risk factors associated with postoperative RT. LASSO regression analysis identified eight independent predictors: maximum sagittal diameter, maximum coronal diameter, age, Knosp grade, ICA invasion, sphenoid sinus pneumatization, tumor consistency, and retrosellar invasion (Figure 1).

**FIGURE 1:**
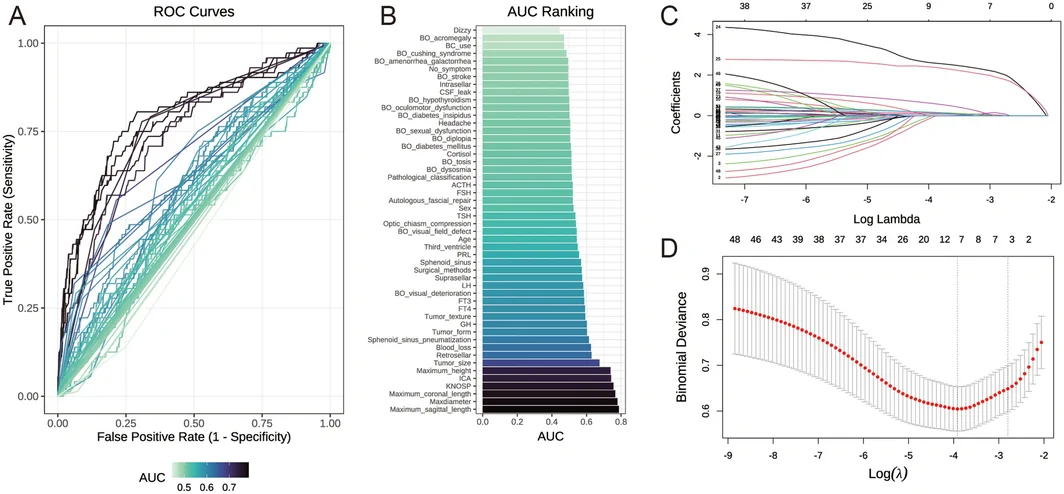
Feature selection and model performance for predicting postoperative residual tumor in PitNETs. (A) ROC curves of candidate variables, with the corresponding AUC values indicated by the color gradient. (B) AUC ranking of individual clinical, radiological, and pathological variables, showing their relative discriminative ability. (C) LASSO regression coefficient profiles plotted against the log(*λ*) sequence. Each colored line represents the trajectory of a variable coefficient as *λ* changes. (D) Ten‐fold cross‐validation curve for the LASSO regression model. The *y*‐axis represents binomial deviance, and the two vertical dashed lines indicate the optimal values of log(*λ*) corresponding to the minimum deviance and the 1‐standard error (1‐SE) criterion.

### 3.5 Model Establishment and Performance

Nine machine learning models were constructed using fivefold cross‐validation. In the training set, RF and SVM both showed high discrimination (RF: AUC = 0.926, 95% CI: 0.866–0.970; SVM: AUC = 0.929, 95% CI: 0.877–0.967) (Figure 2A). In the test set, RF achieved the highest AUC (0.945, 95% CI: 0.860–0.999), whereas XGBoost (AUC = 0.932, 95% CI: 0.845–0.992) and LightGBM (AUC = 0.932, 95% CI: 0.835–0.997) also performed well (Figure 2B). The AUC of RF was significantly higher than that of k‐Nearest Neighbor, Linear Discriminant Analysis, Stacking, and Artificial Neural Network, but was not significantly different from LightGBM (*p* = 0.108), SVM (*p* = 0.726), or XGBoost (*p* = 0.063) in the test set (Delong's test; Table 2). Calibration analysis showed that LightGBM had the lowest Brier score in the test set (0.047), followed by SVM (0.058) and RF (0.064), indicating better probability calibration with LightGBM despite similar discrimination (Figure 2F). Overall, RF achieved the highest validation accuracy (0.938), specificity (0.986), AUC (0.945), and F1 score (0.784). Decision curve analysis suggested a favorable net benefit for RF across clinically relevant threshold probabilities, comparable to LightGBM in several ranges (Figure 2C,D). RF was selected as the final model because of its robust overall performance and interpretability; however, its performance was comparable rather than statistically superior to all advanced algorithms (Figure 3). As a conventional comparator, logistic regression yielded a test‐set AUC of 0.715 (95% CI, 0.598–0.812) and a Brier score of 0.105 (Table S3). Decision curves for logistic regression and RF are shown in Figure S1. For RF, precision and recall were calculated with RT as the positive class in the held‐out test set, yielding an RT recall of 92.3% and precision of 70.6% (Table S4). In RF subgroup analyses, AUCs were 0.850 for functional PitNETs and 0.927 for nonfunctional PitNETs. The corresponding values were 0.725 in giant adenomas and 0.717 in non‐giant adenomas (Table S5).

**FIGURE 2:**
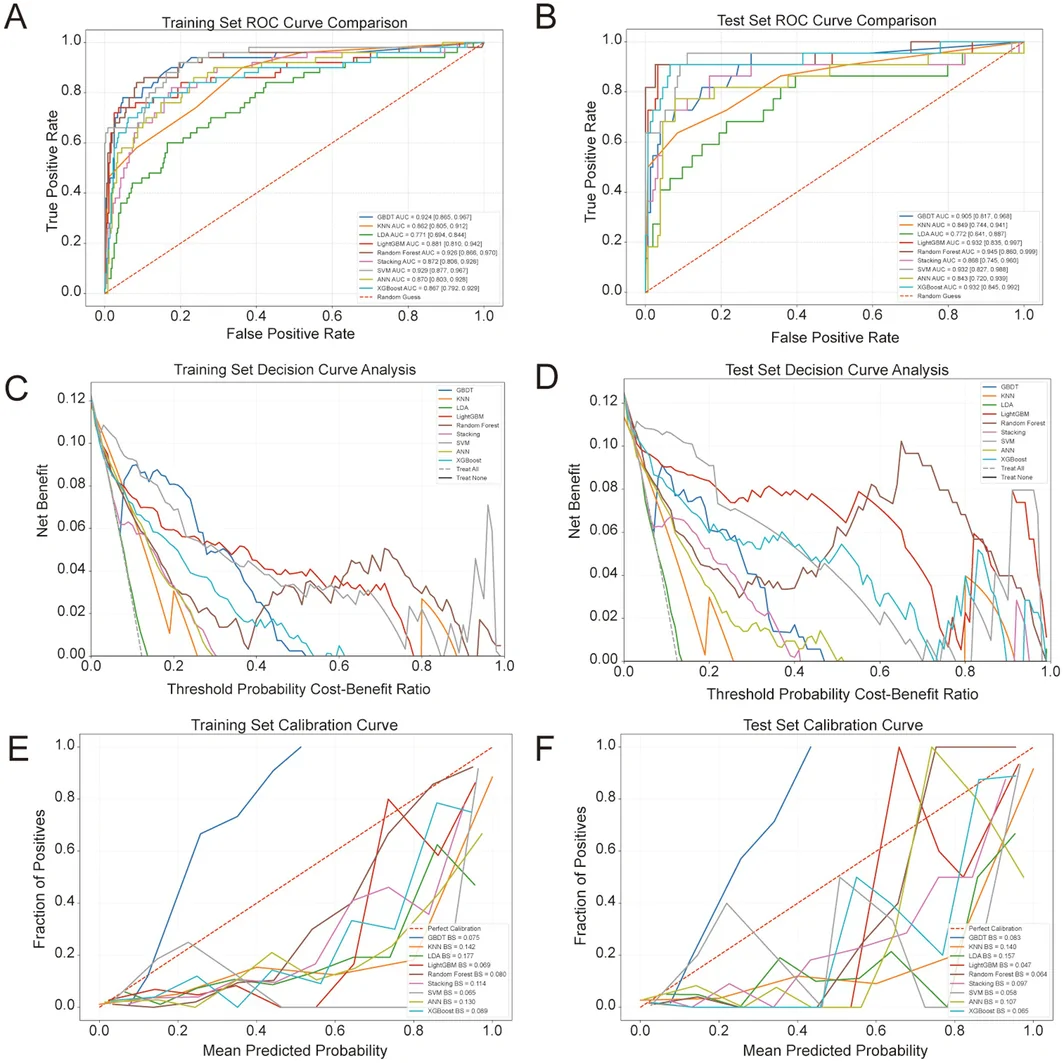
Model performance comparison between training and test sets. (A, B) ROC curves of different machine learning models in the training set (A) and test set (B), with corresponding AUC values indicated. (C, D) DCA for the training set (C) and test set (D), showing the net clinical benefit across a range of threshold probabilities. (E, F) Calibration curves of different models in the training set (E) and test set (F), comparing predicted probabilities with actual observed outcomes.

**TABLE 2:** DeLong test for machine learning models in the test set.

Model 1	Model 2	AUC1	AUC2	Difference of AUC	*Z* statistic	*p*
GBDT	KNN	0.9045	0.8493	0.0552	1.4992	0.134
GBDT	LDA	0.9045	0.7713	0.1333	2.8978	0.004*
GBDT	LightGBM	0.9045	0.9321	−0.0276	−0.7693	0.442
GBDT	RF	0.9045	0.9454	−0.0409	−1.3213	0.186
GBDT	Stacking	0.9045	0.8684	0.0362	0.9859	0.324
GBDT	SVM	0.9045	0.9318	−0.0273	−1.1040	0.270
GBDT	ANN	0.9045	0.8442	0.0604	1.3497	0.177
GBDT	XGBoost	0.9045	0.9321	−0.0276	−0.8152	0.415
KNN	LDA	0.8493	0.7713	0.0781	1.8048	0.071
KNN	LightGBM	0.8493	0.9321	−0.0828	−2.6897	0.007*
KNN	RF	0.8493	0.9454	−0.0961	−3.2241	0.001*
KNN	Stacking	0.8493	0.8684	−0.0190	−0.5474	0.584
KNN	SVM	0.8493	0.9318	−0.0825	−1.8928	0.058
KNN	ANN	0.8493	0.8442	0.0052	0.1221	0.903
KNN	XGBoost	0.8493	0.9321	−0.0828	−2.7041	0.007*
LDA	LightGBM	0.7713	0.9321	−0.1609	−3.7201	< 0.0001*
LDA	RF	0.7713	0.9454	−0.1741	−4.0045	< 0.0001*
LDA	Stacking	0.7713	0.8684	−0.0971	−2.4359	0.015*
LDA	SVM	0.7713	0.9318	−0.1606	−2.6905	0.007*
LDA	ANN	0.7713	0.8442	−0.0729	−1.4660	0.143
LDA	XGBoost	0.7713	0.9321	−0.1609	−3.7991	< 0.0001*
LightGBM	RF	0.9321	0.9454	−0.0133	−1.6053	0.108
LightGBM	Stacking	0.9321	0.8684	0.0638	2.9160	0.004*
LightGBM	SVM	0.9321	0.9318	0.0003	0.0068	0.995
LightGBM	ANN	0.9321	0.8442	0.0880	3.2325	0.001*
LightGBM	XGBoost	0.9321	0.9321	0.0000	0.0000	1.000
RF	Stacking	0.9454	0.8684	0.0770	3.0538	0.002*
RF	SVM	0.9454	0.9318	0.0136	0.3505	0.726
RF	ANN	0.9454	0.8442	0.1012	3.2544	0.001*
RF	XGBoost	0.9454	0.9321	0.0133	1.8559	0.063
Stacking	SVM	0.8684	0.9318	−0.0635	−1.4134	0.158
Stacking	ANN	0.8684	0.8442	0.0242	0.8102	0.418
Stacking	XGBoost	0.8684	0.9321	−0.0638	−2.5353	0.011*
SVM	ANN	0.9318	0.8442	0.0877	1.7892	0.074
SVM	XGBoost	0.9318	0.9321	−0.0003	−0.0069	0.995
ANN	XGBoost	0.8442	0.9321	−0.0880	−2.8641	0.004*

* Denotes a statistically significant difference.

**FIGURE 3:**
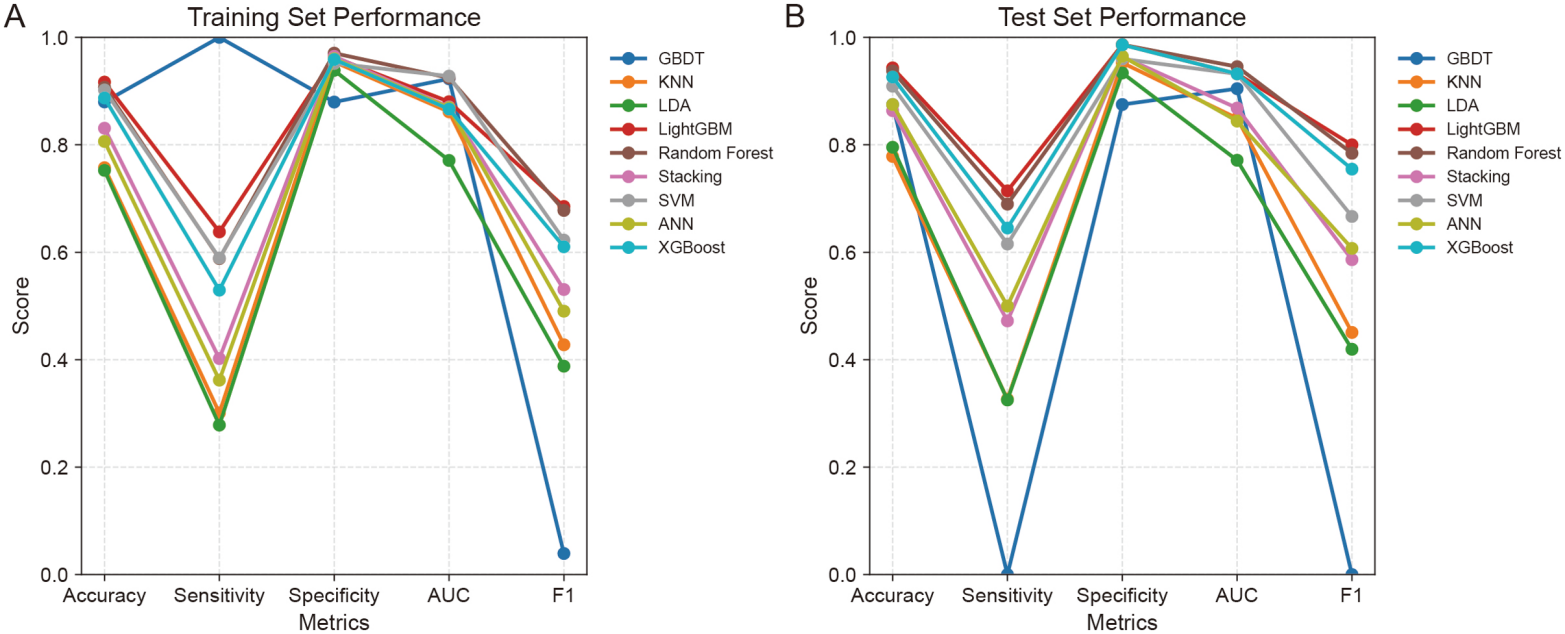
Classification performance of machine learning models for predicting postoperative residual tumor in the training and test sets. Parallel line graph of the evaluation metrics including accuracy, sensitivity, specificity, AUC, and F1‐score for each model in the training set (A) and test set (B). Performance is quantified on a scale from 0.0 to 1.0 for all metrics. Higher values indicate better predictive performance.

### 3.6 Model Interpretation

To improve the transparency, SHAP analysis was performed to quantify the contribution of each predictor to postoperative RT prediction. After recalculating SHAP values using the original non‐SMOTE training samples as the background dataset, the expected model output was 0.271. At the global level, maximum sagittal diameter ranked highest in the SHAP analysis of this internally evaluated RF model, followed by maximum coronal length, age, Knosp grade, ICA invasion, sphenoid sinus pneumatization, tumor texture, and retrosellar invasion (Figure 4A). The SHAP summary plot further demonstrated that larger sagittal and coronal tumor diameters were generally associated with positive SHAP values, indicating an increased predicted probability of postoperative RT (Figure 4B). Higher Knosp grade and ICA invasion tended to shift the model output toward a higher risk prediction, whereas sphenoid sinus pneumatization, tumor texture, and retrosellar invasion had smaller but still measurable effects.

**FIGURE 4:**
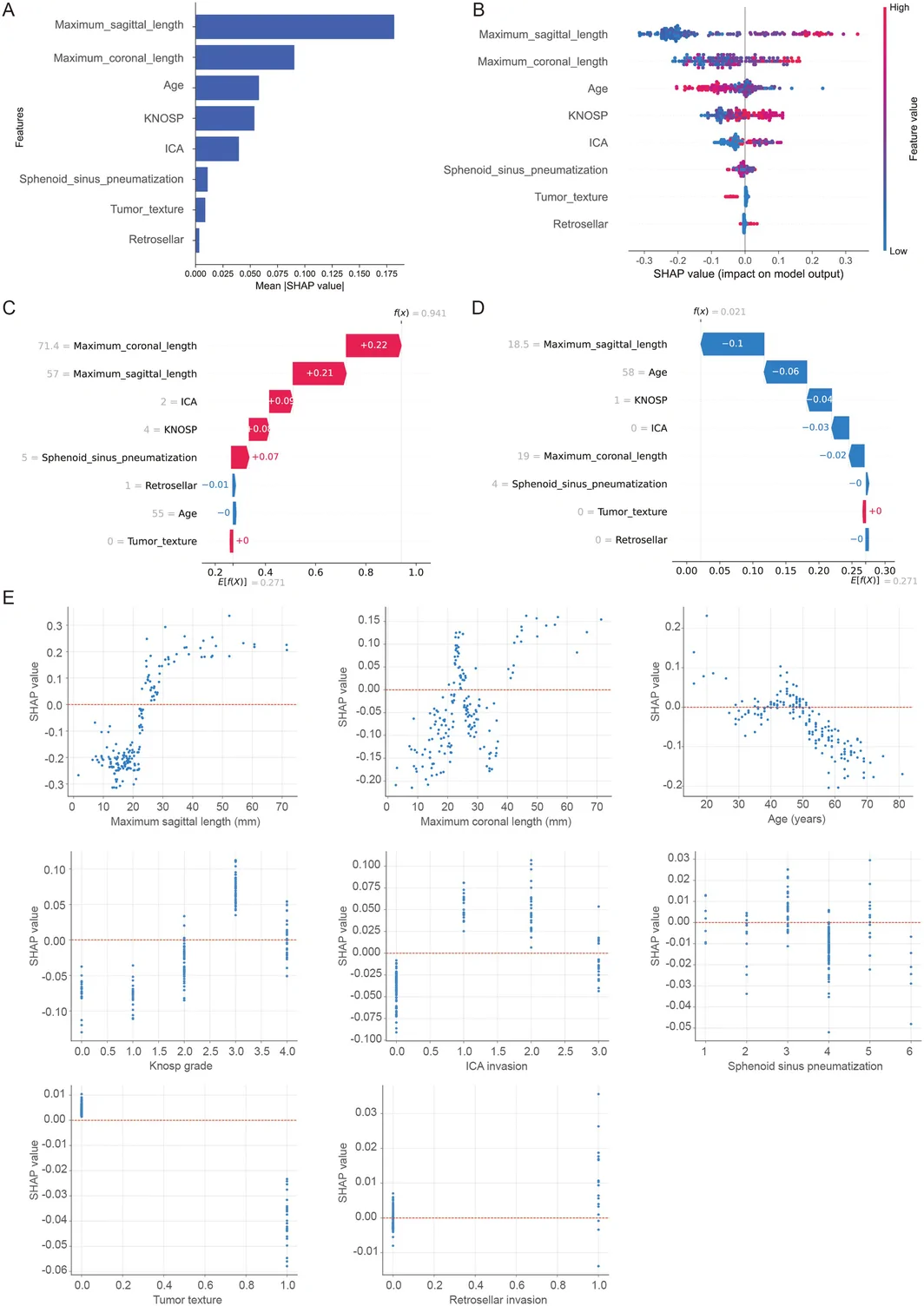
Global and local model explanation by the SHAP method. (A) SHAP summary bar plot of key radiological and clinical features included in the model. (B) SHAP summary dot plot. Each patient has one dot on the line for each feature. The colors of the dots demonstrate the actual values of the features for each patient, as red means a higher feature value and blue means a lower feature value. (C, D) SHAP waterfall plot. Two representative cases demonstrating specific input values for predictive features, including maximum sagittal and coronal lesion dimensions, Knosp grade, ICA involvement, patient age, and categorical imaging characteristics. Red bars indicate features that contribute positively to the prediction, while blue bars indicate negative contributions. (E) SHAP dependence plot. Each dependence plot shows how a single feature affects the output of the prediction model, and each dot represents a single patient. Positive SHAP values increase the model's predicted probability of postoperative residual tumor.

Local SHAP waterfall plots provided individualized explanations for model prediction. In a representative high‐risk case, the predicted probability increased from a baseline value of 0.271 to 0.941. The main positive contributors were a large maximum coronal length, a large maximum sagittal length, ICA invasion, higher Knosp grade, and sphenoid sinus pneumatization, whereas age, tumor texture, and retrosellar invasion contributed minimally or slightly negatively (Figure 4C). Conversely, in a representative low‐risk case, the predicted probability decreased from 0.271 to 0.021. In this patient, smaller sagittal and coronal diameters, older age, low Knosp grade, and absence of ICA invasion were the major factors driving the model toward a low‐risk prediction (Figure 4D). These findings indicate that the model's individual‐level decisions were mainly driven by tumor size and parasellar invasion‐related features, consistent with their global importance rankings.

SHAP dependence plots further revealed nonlinear relationships between individual predictors and model output (Figure 4E). The SHAP value of maximum sagittal length increased markedly when the sagittal diameter exceeded approximately 20–30 mm, suggesting a threshold‐like increase in RT risk with increasing anteroposterior tumor extension. Maximum coronal length also showed an overall positive association with risk contribution, although with greater dispersion. Age displayed a nonlinear effect, with younger patients generally contributing more positively to predicted risk, whereas older age tended to shift predictions downward. For categorical radiological variables, higher Knosp grade and the presence of ICA invasion were associated with increased SHAP values. In contrast, sphenoid sinus pneumatization, tumor texture, and retrosellar invasion showed smaller absolute SHAP values, suggesting that these factors provided additional but less dominant predictive information.

### 3.7 Online Tool Development

To facilitate clinical application, the final RF model was deployed as an interactive web‐based prediction tool (https://pitnet‐predictor.streamlit.app/). Clinicians can input the eight selected predictors, including age, maximum coronal length, maximum sagittal length, retrosellar invasion, ICA invasion, Knosp grade, sphenoid sinus pneumatization, and tumor texture, to obtain an individualized predicted probability of postoperative RT. The platform also generates a SHAP force plot to visualize how each variable contributes to the final prediction. When representative patient‐specific parameters were entered into the model, the tool predicted a 94.47% probability of postoperative RT (Figure 5). The corresponding SHAP force plot shows that the prediction was mainly driven upward by younger age, larger maximum sagittal length, and sphenoid sinus pneumatization.

**FIGURE 5:**
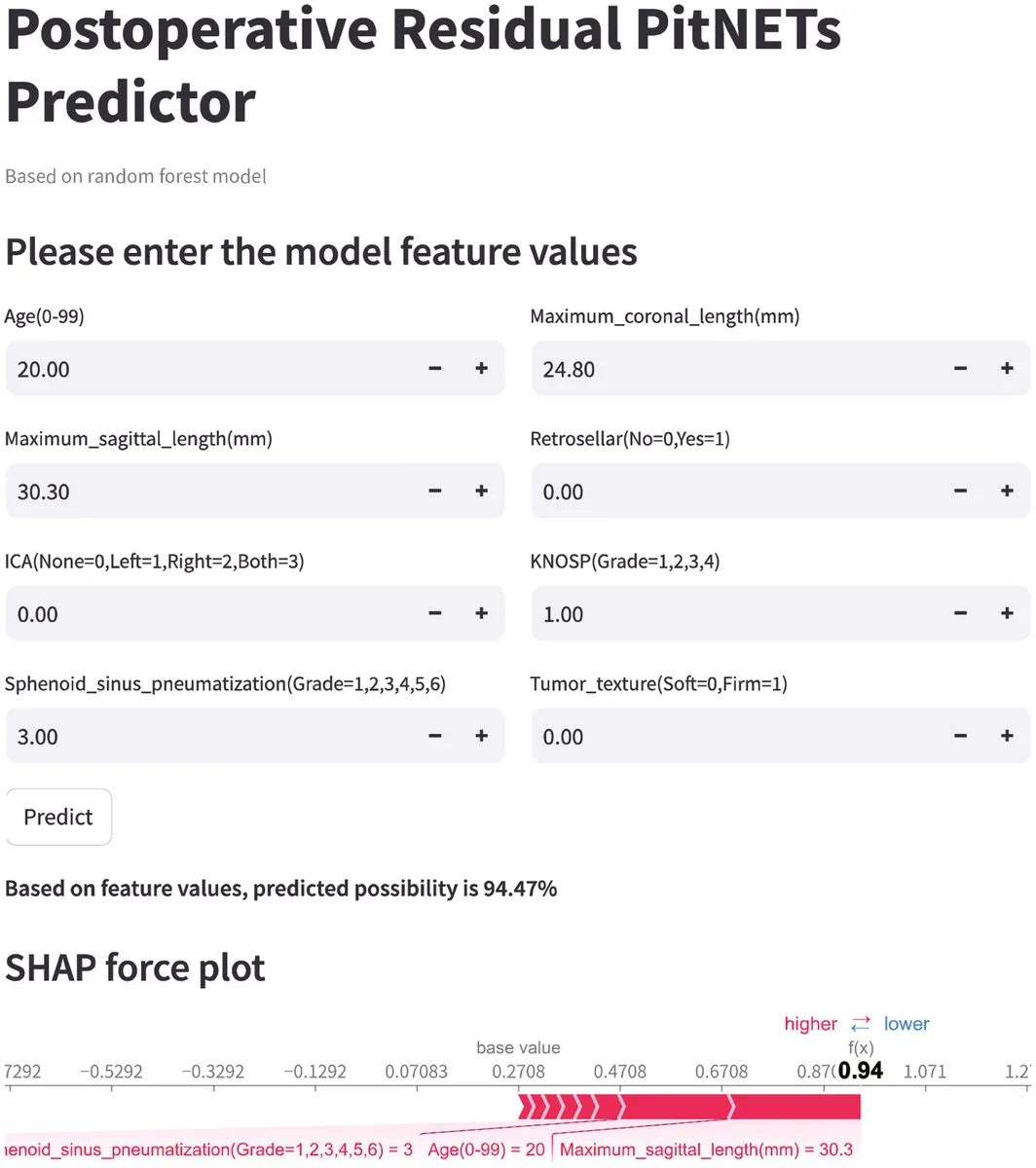
Web‐based tool prediction and SHAP force plot visualization. The eight‐feature RF model is available as a web‐based tool for predicting postoperative residual tumor in patients with PitNETs. After users enter eight clinical and radiological variables, the tool calculates the predicted probability of postoperative residual tumor. A SHAP force plot illustrates the contribution of each variable to the individual prediction, with red bars indicating positive effects and blue bars indicating negative effects. In the presented case, the predicted probability of postoperative residual tumor was 94.47%, underscoring the need for meticulous preoperative planning and close postoperative follow‐up.

## 4 Discussion

We developed and internally assessed an ML model for radiologically confirmed postoperative RT in a single‐center PitNET cohort. A series of clinical and radiological variables were included, and among nine candidate algorithms, RF showed strong internal discrimination, with an AUC comparable to LightGBM, XGBoost, and SVM. SHAP analysis identified tumor size and Knosp grade as influential predictors within this cohort.

PitNETs account for approximately 15% of all primary intracranial tumors [1, 20]. Although most PitNETs are benign, their heterogeneity and the complex anatomy of the skull base contribute to substantial surgical challenges. Tumors may extend superiorly into the third ventricle, posteriorly into the retrosellar region, anteriorly to the planum sphenoidale, or laterally into the cavernous sinus, greatly increasing the difficulty of complete resection. More than 30% of PitNETs exhibit invasive behavior with infiltration into surrounding structures, Raverot et al. reported that 42% of patients experienced tumor recurrence or progression during follow‐up [21]. Therefore, preoperative prediction of the likelihood of postoperative residual tumor and the design of appropriate follow‐up and sequential treatment strategies are particularly important for improving patient prognosis and quality of life. Several classification and predictive systems have been developed to assess the extent of surgical resection for PitNETs [22, 23, 24, 25, 26, 27, 28]. Patel et al. constructed models incorporating maximum tumor diameter and morphology [25], and Fiore et al. developed a modified TRANSSPHER grading system for non‐functioning pituitary adenomas, achieving an AUC of 0.85 [26]. However, most studies have relied on preoperative imaging variables, often lacked clear separation of training and testing cohorts, suffered from insufficient sample sizes, and presented limited real‐world clinical applicability, restricting predictive accuracy and generalizability [28].

Importantly, the predictive variables used in our model can be readily obtained from electronic medical records, making it highly feasible for clinical application. Compared with previous research on predicting postoperative residual tumor in PitNETs, our study incorporated conventional imaging features and systematically integrated clinical symptoms, pathological characteristics, and hormonal levels, encompassing a total of 50 candidate variables. This multidimensional approach provides a more comprehensive representation of tumor biology and surgical risks, enhancing clinical utility and generalizability. Moreover, a rigorous feature selection strategy was employed: initial univariate regression identified relevant predictors, reducing noise, followed by LASSO regression for variable shrinkage and selection, ensuring focus on the most valuable predictors. This two‐step approach effectively mitigated overfitting, ultimately retaining eight variables for model construction: maximum sagittal diameter, maximum coronal diameter, patient age, Knosp grade, ICA invasion, sphenoid sinus pneumatization, tumor texture, and retrosellar extension.

By integrating routinely available clinical and radiological variables, the RF model demonstrated strong discrimination and favorable clinical utility. However, DeLong testing in the test set indicated that RF was statistically comparable to LightGBM, XGBoost, and SVM in terms of AUC; therefore, RF should be interpreted as a well‐performing final model rather than a model with proven superiority. An additional issue highlighted by the present analysis is the tradeoff between discrimination and calibration. Although RF achieved the numerically highest test‐set AUC and F1 score, its Brier score (0.064) was higher than that of LightGBM (0.047), indicating less accurate probability calibration. This distinction is clinically important because a model with excellent discrimination can still overestimate or underestimate absolute risk. In preoperative counseling and postoperative surveillance planning, RF outputs should be interpreted primarily as individualized risk stratification rather than as calibrated probabilities.

Compared with the logistic regression benchmark, RF showed a numerically higher AUC and lower overall prediction error in the internal test set. The class‐specific analysis clarifies the practical trade‐off: most RT cases were identified, while approximately three in ten positive predictions were false positives. Predicted risk may therefore guide counseling and follow‐up planning, while postoperative imaging remains necessary to establish RT.

One of the key innovations of this work lies in the integration of explainable artificial intelligence. While machine learning models are often criticized as “black boxes,” we applied SHAP analysis to provide both global and patient‐specific interpretability. At the global level, sagittal and coronal tumor diameters, Knosp grade, ICA invasion, and age had relatively large SHAP contributions in the internally evaluated RF model. At the individual level, waterfall plots illustrated how each feature contributed to the predicted risk. This transparent framework enhances clinical trust and facilitates personalized decision‐making.

The corrected SHAP baseline also requires careful interpretation. The baseline probability of 0.271 was higher than the observed RT rate of 12.3%, mainly because SHAP expected values reflect the average predicted output of the trained model on the chosen background dataset, rather than the empirical event rate. In addition, the model was trained with a class‐imbalance strategy and tree‐based probability estimates, which may shift the mean predicted probability even when the SHAP background samples are not oversampled. Thus, the waterfall plots should be used to understand how individual variables move a risk estimate relative to the model baseline, while absolute probability calibration should be assessed separately using calibration curves and Brier scores.

In this single‐center cohort, tumor dimensions and indicators of parasellar invasion contributed to RF predictions of postoperative RT. Maximum sagittal diameter had a relatively large SHAP contribution, alongside coronal diameter and Knosp grade, in the internally evaluated model. Park et al. also reported that the anteroposterior diameter of the suprasellar component is a critical driver of surgical resectability, with larger diameters associated with higher risks of postoperative CSF leak and complications, suggesting that tumor anteroposterior diameter should be incorporated into grading systems traditionally based only on cavernous sinus invasion [29]. Similarly, Hannan et al. demonstrated that including AP diameter in preoperative assessment improved gross‐total resection rate predictions. In transsphenoidal surgery, tumors with large anteroposterior dimensions may fail to descend from the suprasellar region into the sellar, increasing the risk of cerebrospinal fluid leak or hemorrhage due to excessive traction [30, 31]. This technical limitation often prompts more conservative surgical strategies and increases the likelihood of residual tumor [32]. Similarly, tumor lateral extension significantly impacts surgical outcomes. A larger maximum coronal diameter is often associated with greater parasellar invasion and technical difficulty. Knosp classification, based on the relationship between pituitary adenomas and the ICA [33], is not only valuable in assessing tumor invasiveness but also plays an essential role in predicting residual and recurrent disease [34, 35]. Gross‐total resection rates decrease with increasing Knosp grade, falling from 55.8% in Grade 3A to 30% in Grade 3B [34]. Multiple studies have confirmed that greater parasellar invasion correlates with reduced rates of endocrine remission and gross‐total resection [36, 37, 38], which aligns with our findings. Endoscopic transsphenoidal surgery, now the preferred approach for most PitNETs, offers a wider lateral field of view compared to the microscopic approach [1], and a binostril endoscopic approach provides greater maneuverability than a mononostril approach. However, lateral visualization and instrument depth remain limited by nasal anatomy [39]. For tumors with large coronal diameters, blind spots may persist, increasing the risk of residual tumor. Larger lateral dimensions are also associated with cavernous sinus and ICA invasion, leading surgeons to adopt more conservative resection strategies to avoid complications [36, 40]. In addition, large tumor volume is linked with increased intraoperative bleeding, which further compromises visualization and increases the risk of residual disease [37].

We also found that retrosellar extension was associated with postoperative residual tumor, consistent with the findings of Juraschka et al. [41] The degree of sphenoid sinus pneumatization directly influences the surgical window width of the transnasal–transsphenoidal approach, the relative distance to critical neurovascular structures, and the accessibility of the cavernous sinus region [42]. These factors, in turn, alter the boundaries of surgical accessibility, visibility, and maneuverability, thereby impacting the extent of tumor resection and the risk of residual disease. Such anatomical variations provide valuable reference for surgical strategy formulation [43]. In our model, this variable was incorporated and demonstrated relatively high explanatory power, suggesting the potential value of explicitly integrating anatomical corridor factors into risk assessment.

Our study additionally highlights clinical applicability and translational potential. All variables incorporated into the model are routinely available from standard clinical and radiological assessments, without the need for advanced radiomics or molecular profiling. Importantly, we deployed the RF model into an interactive online platform, enabling clinicians to enter patient‐specific data and instantly receive individualized risk predictions alongside SHAP‐based visual explanations. As shown in the case of Figure 6A, preoperative MRI revealed a typical giant adenoma with suprasellar extension. After inputting the parameters, the model output indicated a high‐risk individualized prediction probability. Early postoperative contrast‐enhanced MRI still demonstrated focal enhancement, suggesting residual tumor (Figure 6B). The SHAP force plot of the model showed that the main contributors driving the “high‐risk” judgment for this patient included a larger maximum sagittal diameter (30.3 mm), younger age (20 years), and the sphenoid sinus pneumatization subtype (semisellar type) (Figure 6C). Correspondingly, in Figure 6D, an elderly patient is presented whose tumor showed a similar extent of parasellar invasion and a comparable suprasellar anteroposterior diameter to the previously described case. The overall tumor morphology was also similar; however, the predicted probability of residual tumor was much lower (16%). Postoperative MRI confirmed the absence of residual tumor (Figure 6E). From the perspective of follow‐up and postoperative management, if the model had predicted high risk preoperatively, a more aggressive surgical strategy (such as extended approaches or staged surgery) could have been considered intraoperatively, while enhanced imaging surveillance and early adjuvant therapy (such as stereotactic radiotherapy) should be implemented postoperatively. This represents the critical point where ML models can improve patient outcomes in real‐world practice. At the same time, this integration of predictive accuracy, interpretability, and clinical accessibility represents a meaningful step toward precision neurosurgery in PitNETs.

**FIGURE 6:**
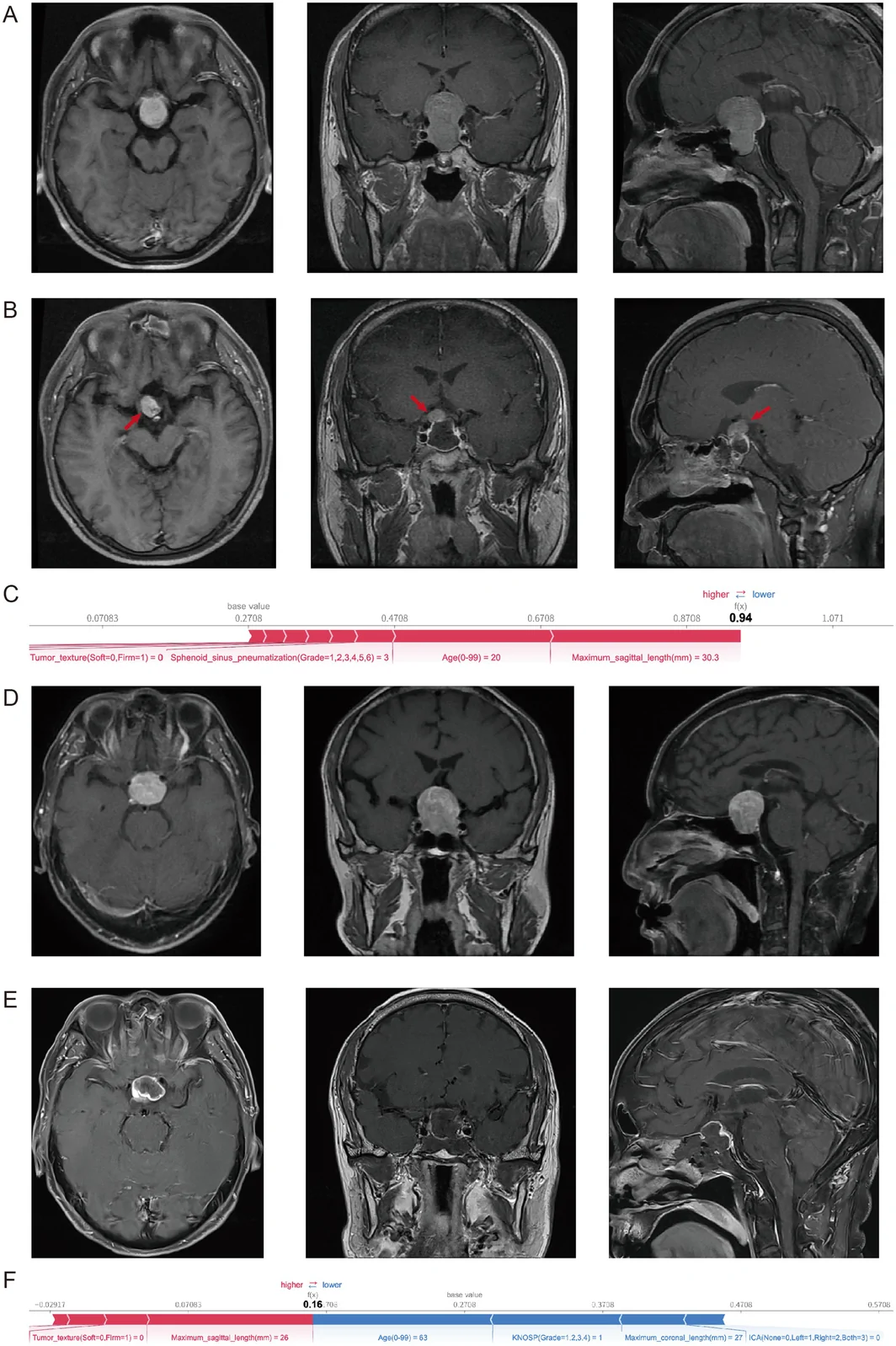
Preoperative and postoperative MRI with SHAP‐based individualized predictions of postoperative residual tumor. (A) Preoperative contrast‐enhanced magnetic resonance imaging (MRI) of Case 1, shown in axial, coronal, and sagittal views. (B) Postoperative contrast‐enhanced MRI of Case 1, with the residual tumor indicated by the red arrow. (C) SHAP force plot of the machine learning model for Case 1. Red bars shifting to the right of the baseline indicate variables contributing to an increased individualized risk of postoperative residual tumor. The predicted probability of postoperative residual tumor was 0.94. (D) Preoperative contrast‐enhanced MRI of Case 2. (E) Postoperative contrast‐enhanced MRI of Case 2, demonstrating gross total resection. (F) SHAP force plot for Case 2, showing a low predicted probability of postoperative residual tumor, which is consistent with the postoperative imaging findings.

This study had several limitations. Early postoperative MRI within 48 h may occasionally overestimate RT because postoperative hematoma, inflammatory enhancement, packing materials, and reactive tissue changes can mimic true RT. To minimize this bias, our final RT classification was not based solely on 48‐h MRI but was confirmed by longitudinal follow‐up MRI. In our sensitivity analysis, the agreement between 48‐h MRI and final longitudinal confirmation was high. Nevertheless, because this was a retrospective study and follow‐up intervals were not completely uniform, future prospective studies with standardized early and delayed postoperative MRI protocols are warranted. Second, model development and evaluation relied on a random split and cross‐validation within one center, without temporal or external validation. Patients in the internal subsets shared imaging protocols, surgical practice, and case mix, so the internal performance estimate may overstate performance in other settings. Selecting the final model after comparing nine algorithms on internal results may introduce additional selection‐related optimism. Generalizability to other centers and future patient cohorts remains unproven; independent temporal and multicenter cohorts should reassess discrimination, calibration, and net benefit. Subgroup estimates were also imprecise because each stratum contained only 7–19 RT cases. Additionally, due to the 2022 World Health Organization update on PitNETs pathological classification, some molecular marker data were incomplete in our cohort and therefore not included. Incorporating these molecular markers in future studies may further enhance predictive performance. Third, although several grading or scoring systems for pituitary tumor resectability have been proposed, direct comparison was limited by differences in target populations, endpoint definitions, required variables, and the lack of publicly available code or individual‐level datasets for many models. Therefore, the present model should not be interpreted as having demonstrated superiority over existing scoring systems. Rather, it provides an explainable and web‐accessible complementary tool that integrates multiple routinely available variables. Prospective external validation against established grading systems, such as Knosp‐based, TRANSSPHER‐derived, and other imaging‐based scores, will be necessary to define its incremental clinical value.

## 5 Conclusion

We developed an explainable ML model to predict radiologically confirmed postoperative RT in PitNET patients using routinely available clinical and radiological variables. The RF model showed strong internal validation performance and was selected as the final web‐based model, while its AUC was comparable to LightGBM, XGBoost, and SVM rather than statistically superior to all advanced algorithms. The online tool may support risk discussions, but its performance in other centers and future patient cohorts remains unverified. Independent temporal and multicenter validation, including calibration assessment and comparison with established scores, is needed before clinical use.

## Author Contributions

Q.L., G.T., and Y.L. offered direction and guidance for the manuscript. M.L. and C.Z. were responsible for data collection. M.L. drafted the manuscript and illustrated the figures and tables for the manuscript. M.L. and C.Z. contributed to the revision of the manuscript. All the authors approved the final manuscript.

## Funding

This study was supported by two grants from the National Natural Science Foundation of China (Nos. 82472987 and 82172834), and the National Key R&D Program of China (No. 2022YFE0133400).

## Ethics Statement

The study was conducted in accordance with the Declaration of Helsinki, and approved by the Ethics Committee of Xiangya Hospital (2024030156). Given the retrospective nature of this study and anonymization of patient information, informed consent was waived by the Ethics Committee.

## Conflicts of Interest

The authors declare no conflicts of interest.

## Supporting information


**Figure S1:** Decision curve analysis for predicting postoperative residual tumor. The *x*‐axis shows threshold probability, and the *y*‐axis shows net benefit. The blue and brown curves represent the logistic regression and random forest (RF) models. The gray dashed and black horizontal lines represent the treat‐all and treat‐none reference strategies.

**Table S1:** Agreement between 48‐h MRI‐based assessment and final longitudinal follow‐up confirmation of postoperative residual tumor.

**Table S2:** Interobserver agreement for key radiological predictors.

**Table S3:** Predictive performance of baseline logistic regression and random forest models in the training and test cohorts.

**Table S4:** Precision and recall of the random forest model for postoperative residual tumor prediction in the training and held‐out test cohorts.

**Table S5:** Subgroup performance of the random forest model by functional status and tumor size in the held‐out test set.

## Data Availability

The code used in this study is publicly available at https://github.com/cyhwqhm/RF2. The raw data generated and/or analyzed during the current study are available from the corresponding author on reasonable request.
