# Supplementary material for: Multiple Machine Learning Algorithms for Predicting Postoperative Residual Tumor in Pituitary Neuroendocrine Tumors
Source: CNS Neurosci Ther. 2026 Nov 2;32(11):e71244. doi: 10.1002/cns.71244 (PMC13728462; doi:10.1002/cns.71244)
Supplement: Supplementary file 1 [file CNS-32-e71244-s001.pdf]

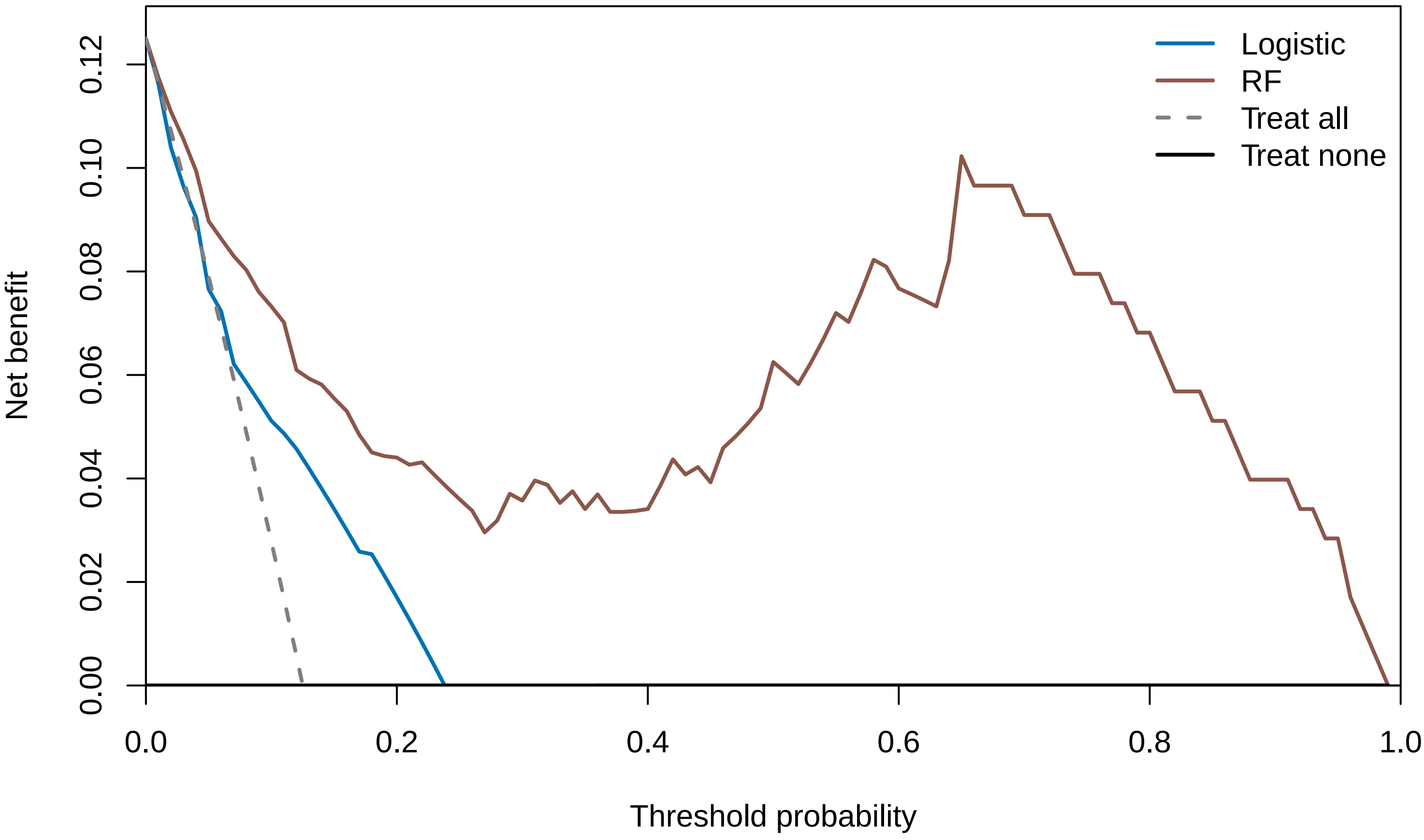
Logistic
RF
Treat all
Treat none
Net benefit
0.00
0.02
0.04
0.06
0.08
0.10
0.12
0.0
0.2
0.4
0.6
0.8
1.0
Threshold probability
